# Are vertical jumps able to predict 24-month follow-up functional geriatric assessment in a healthy community-dwelling older cohort?

**DOI:** 10.1007/s40520-022-02230-9

**Published:** 2022-09-02

**Authors:** Rebecca Diekmann, Sandra Hellmers, Sandra Lau, Andrea Heinks, Lena Elgert, Juergen M. Bauer, Tania Zieschang, Andreas Hein

**Affiliations:** 1 Assistance Systems and Medical Device Technology, Department of Health Services Research, School of Medicine and Health Sciences, Carl von Ossietzky University of Oldenburg, Ammerlaender Heerstr. 140, 26129 Oldenburg, Germany; 2 Geriatric Medicine, Agaplesion Bethanien Krankenhaus Heidelberg, Ruprecht-Karls-University, Heidelberg, Germany; 3 Peter L. Reichertz Institute for Medical Informatics of TU Braunschweig, Hannover Medical School (MHH), Hannover, Germany; 4 Geriatric Medicine, Department of Health Services Research, Carl von Ossietzky University of Oldenburg, Oldenburg, Germany

**Keywords:** Geriatric medicine, Physical performance, Falls, Muscle strength, Prevention

## Abstract

**Background:**

When older adults fall below the thresholds of functional geriatric assessment (FGA), they may already be at risk of mobility impairment. A reduction in (jumping) power could be an indication of functional decline, one of the main risk factors for falls.

**Objective:**

This paper explores whether six-month delta (∆) values of muscle power can predict 24-month follow-up FGA in older adults.

**Methods:**

This observational study of independent, healthy, high-performing community-dwelling adults aged 70 + years involved FGA (mobility, balance, and endurance tests) at baseline (*t*_0_), after 6 months (*t*_1_), and after 24 months (*t*_2_); maximum jumping power (max JP) was determined at *t*_0_ and *t*_1_. A predictive linear model was developed in which the percentage change of Δmax JP_0,1_ was transferred to all FGA (*t*_0_) values. The results were compared with measured FGA values at *t*_2_ via sensitivity and specificity in terms of the clinically meaningful change (CMC) or the minimal detectable change (MDC).

**Results:**

In 176 individuals (60% female, mean age 75.3 years) the mean percentage (SD) between predicted and measured FGA ranged between 0.4 (51.3) and 18.11 (51.9). Sensitivity to identify the CMC or MDC of predicted FGA tests at *t*_2_ ranged between 17.6% (Timed up and go) and 75.0% (5-times-chair-rise) in a test-to-test comparison and increased to 97.6% considering clinically conspicuousness on global FGA.

**Conclusion:**

The potential of jumping power to predict single tests of FGA was low regarding sensitivity and specificity of CMC (or MDC). 6 months Δmax JP seem to be suitable for predicting physical function, if the measured and predicted tests were not compared at the test level, but globally, in the target group in the long term.

**Supplementary Information:**

The online version contains supplementary material available at 10.1007/s40520-022-02230-9.

## Background

Preventive measures to help maintain physical function are a crucial factor in enabling older adults to retain their independence and enjoy a high quality of life. Vulnerable groups that may benefit from preventive interventions and exercises are often identified via functional geriatric assessment (FGA). When older adults fall below established thresholds in these assessments, they may be at greater risk of negative events such as falls. Neri et al. demonstrated a threefold increased risk of falls in 195 older women (mean age 68 years) with low handgrip strength [1]. Nevertheless, a relevant deterioration of physical performance in fit older adults occurs regardless of geriatric thresholds [2]. To enable early intervention with the target group of older adults aged 70 years or more—in the sense of primary prevention—it is, therefore, particularly important to assess physical performance and identify deterioration at an early stage. According to the International Conference on Frailty and Sarcopenia Research (ICFSR) Task Force, clinical meaningfulness is defined by the requirement of a result to be available assessing something that is clinically important and that has a major impact on how the patient feels, functions, and survives. The identification of clinically meaningful changes in terms of physical function, therefore, appears to have an impact on older people’s health outcome, enabling preventive measures to be taken. Both distribution-based and anchor-based methods were used to determine several similar terms including the minimally clinically important difference (MCID), the clinically meaningful difference (CMD) or the minimally important difference (MID) in physical function in previous research [4, 5].

Both muscle strength and muscle power (power = force*velocity) are important determinants of physical function and mobility skills in older adults [6–8]. These two parameters have a significant effect on older individual’s fear of falling and their quality of life [9]. The assessment of muscle strength and muscle power is highly relevant, particularly in the screening and diagnosis of sarcopenia [8]. Since muscle function predicts disability and mortality better than measurements of muscle mass alone, both measurements are included in definitions of sarcopenia [10]. Muscle power declines earlier than muscle strength due to a reduction of motor unit numbers with aging [11]. Consequently, muscle power tests have the potential to identify functional decline at an early stage. In FGA, muscle power is often determined using sit-to-stand and stair-climbing tests, which are quantified by the number of repetitions or total time (measured conventionally by stop watches) to complete the test. Additionally, vertical jump parameters from the countermovement jump (CMJ) such as jump height and jumping power [12] represent another assessment of muscle power in older adults [13, 14]. In these assessments, force plates are often used [15]. Jump height and hand grip strength were strongly correlated to leg press force according to Pijnapples et al. [16] and Siglinsky et al. suggested that jumping power and jump height potentially indicate a change in muscle function more precisely and at an earlier stage than existing tools [12]. Singh et al. showed significantly lower jumping power in adults suffering from sarcopenia according to low skeletal muscle mass [17]. Runge sought to identify the effect of aging on muscle mass and muscle power and concluded that jumping tests are a promising extension to chair rise tests with regard to the prediction of immobilization and the risk of falls [18]. However, data thus far is mainly based on cross-sectional analyses and has not been confirmed by longitudinal studies.

This paper, therefore, focuses on jumping power as a proxy for muscle power, and its potential to longitudinally predict the functional outcomes of FGA. The objective of this study was to test whether vertical jump parameters with twice (six-month follow-up) repeated measurements of maximum jumping power (Δmax JP_0_,_1_) have the potential to predict functional tests of functional geriatric assessment (GCA *t*_2_) within 2 years in healthy community-dwelling older adults.

## Methods

A cohort of community-dwelling, healthy adults aged 70 years or more participated in this longitudinal observational study. FGA was conducted at baseline (*t*_0_), after 6 months (*t*_1_), and after 24 (*t*_2_) months. Inclusion criteria were a minimum age of 70 years; community-dwelling; no severe acute diseases (e.g. lung, kidney or heart); no difficulties in climbing a flight of ten steps; the ability to attend assessments independently; no pacemaker or other electronic implants; and a timed up and go test < 20 s. Following a telephone conversation with each participant, the subjects were sent a written informed consent form at least one week before the baseline evaluation. All subjects signed the informed consent form. The study protocol was approved by the Medical Ethics Committee of the Hannover Medical School (MHH) (No. 6948), Germany.

### Physical performance of functional geriatric assessment

FGA covered the measurement of handgrip strength (HGS), the timed up & go test (TUG), the stair climb power test (SCPT (Power)), the short physical performance battery (SPPB), the 5 times chair rise test (5TCR), the 4-m gait speed test (4mGS), and the six-minute walk test (6mWT), as described in detail in Diekmann et al. [2].

### Countermovement jump

For all variables of vertical jumps, a countermovement jump (CMJ) was performed on a ground reaction force platform (AMTI AccuPower, sampling rate 200 Hz) using a personal computer and an integrated analog/digital board and software. The AccuPower allows the measurement of forces in a vertical direction (Fz) in the range of 0–8900 N with a resolution of 4.3 N/bit [due to 12-bit internal AD (± 2048 units)]. Jumps were recorded and analyzed using AccuPower software version 2.0. This system computes the subject’s vertical velocity by integrating the ground reaction force, as described earlier [19]. The instruction given to the subjects was as follows: “Stand in the middle of the force plate with your feet apart at hip width. Bend your knees at an angle of about 90°. When jumping vertically, straighten your knees, being careful not to lift your feet up towards your buttocks. Try to jump as high as you can.” No instructions were given on what to do with the arms. One preliminary jump was permitted. Immediately before starting the test, the subject was asked to stand still to enable their weight to be measured by the force plate. There was a break of one minute between each of the three CMJs. The tester remained close to the subject at all times to ensure safety (e.g. avoiding a fall). The maximum jumping power (max JP) of the subject’s three jumps was selected and recorded in watts. CMJs were performed at *t*_0_ and *t*_1_ only, and not at *t*_2_ for safety reasons. Data were checked for plausibility, and outliers with a minimum one value of ‘zero’ from the three jumps were excluded. Delta was calculated between *t*_0_ and *t*_1_ (Δmax JP_0,1_).

### Linear predictive model

To analyze the predictive potential of max JP_0_,_1_ for 24-month follow-up FGA, the percentage changes of ∆max JP_0,1_ were transferred to all t_0_ tests of FGA.

FGA is a placeholder for all FGA tests performed (HGS, SCPT, TUG, 4mGS, 5TCR, SPPB, 6mWT). This procedure assumes a linear development of the physical functionality of participants.

$${\text{FGA}}\left( {t_2 } \right){\text{pre}}\,{\text{JP}} = {\text{FGA}}\left( {t_0 } \right) + 4 \cdot \,\,\frac{{\left( {\frac{{100\left[ \% \right] \cdot \max {\text{JP}}\left( {t_1 } \right)}}{{\max {\text{JP}}\left( {t_0 } \right)}} - 100\left[ \% \right]} \right) \cdot {\text{FGA}}\left( {t_0 } \right)}}{100\left[ \% \right]}\cdot \,\,\frac{{\left( {\frac{{100\left[ \% \right] \cdot \max {\text{JP}}\left( {t_{1} } \right)}}{{\max {\text{JP}}\left( {t_{0} } \right)}} - 100\left[ \% \right]} \right) \cdot {\text{FGA}}\left( {t_{0} } \right)}}{100\left[ \% \right]}$$

### Statistical analysis

Descriptive data are shown as means (standard deviation SD), minimum, maximum, and interquartile range (25th–75th percentile) for continuous variables and frequencies in absolute numbers (*n*) and in percentages (%) for categorical variables. Data were checked for normal distribution assessed by the Shapiro–Wilk-Test. Pearson’s correlation coefficient was used to measure the correlation of two continuous variables. Percentage delta values of max JP were considered if they had changed more than 1% between *t*_0_ and *t*_1_ to minimize the influence of noise in the sensor-based measurement. The Wilcoxon test was used across two test time points [∆max JP (*t*_0_, *t*_1_)]. A *p*-value of ≤ 0.01 is said to be statistically significant. To be able to evaluate the precision of the value calculated by the predictive model, the % mean values between the measured and the predicted tests of all FGA tests were calculated.

### Clinically meaningful change and minimal detectable change

The clinically meaningful change (CMC) and the minimal detectable change (MDC) for all FGA measurements were identified by literature research and shown in Table 1. Anchor-based and distribution-based methods were used to determine such cut-offs for physical function. In line with Guralnik et al. [4], anchor-based responsiveness was assessed as follows: the participants were grouped according to the self-reported global rating of improvement (better versus no change or worse). The distribution-based responsiveness analysis provided an estimate of the effect size, the minimal detectable change based on a 90% confidence interval (MDC90), and the percentage of participants exceeding MDC90 by a group. Distribution-based methods use statistical and psychometric properties of a measure to estimate the effect size and the standard error of measurement (SEM = *σ*(1 − *r*)1/2, where *σ* = standard deviation and *r* = reliability as functions of variability and reliability. In contrast, anchor-based methods use a change in the patient’s or provider’s perception to identify the corresponding magnitude of change in a selected measure. The standard error of measurement (SEM) was calculated as SD (standard deviation) √1 − ICC (intraclass coefficient); MDC with 90% confidence was calculated as SEM × √2 × 1.65 in each age group. MDC90 is interpreted as the smallest change in a measure that can be considered a real change beyond measurement error with a 90% confidence interval.

**Table 1:** Clinically meaningful change or mini detectable change for all functional assessments

	CMC	MDC	References	Comments
HGS	–	–	[18]	Low HGS was defined according to gender and BMI specific Fried phenotype frailty criteria
SCPT	−	−44 W	[19]	
TUG		2.05 s	[20]	From a cohort of 75 + years
4mGS	−0.10 m/s	–	[21]	
5TCR	–	−3.94 s	[20]	From a cohort of 75 + years
SPPB	−1 Point	–	[21]	
6MWT	−50 m	–	[21]	

*HGS* handgrip strength, *SCPT* stair climb power test, *TUG* timed up and go, *4 m GS* 4-m gait speed, *5 TCR* 5-times-chair-rise, *SPPB* short physical performance battery, *6MWT* six-minute walk test, *W* Watts, *sec* seconds

CMC was prioritized for further analysis, if not available, MDC was accepted. The values presented in Table 1 were used for further analysis. The 4mGS was measured in total time in seconds for the 4 m (see Table 2), to compare the difference from *t*_0_ to *t*_2_ with the CMC of −0.10 m/s, the total seconds were converted into m/s. For all measurement variables, a value below the specified value was defined as clinically relevant, and sensitivity and specificity were determined on the basis of the measured *t*_0_ − t_2_ FGA values.

**Table 2:** The characteristics of those who jumped at all three-time points (*t*_0_, *t*_1_, *t*_2_), in total *n* = 176 (*n* = 106 (60.2%) female)

*N* = 176	Min	Max	Mean (SD)	1st–3rd quartile	*p*-value^$^	*p*-value*
Age (years) *t*_0_	70	87	75.3 (3.7)	72.0–77.0	0.000	0.000
*t*_1_	70	88	75.8 (3.7)	73.0–78.0	0.000	
*t*_2_	72	89	77.3 (3.7)	74.0–79.0	0.000	
BMI (kg/m^2^) *t*_0_	19.2	42.0	27.0 (3.9)	24.1–29.2	0.000	0.691
*t*_1_	19.5	42.4	27.0 (4.0)	24.3–29.1	0.000	
*t*_2_	19.5	40.7	27.0 (3.9)	24.0–28.9	0.000	
Max JP (W) *t*_0_	621.3	3379.5	1827.9 (512.8)	1451.2–2140.6	0.000	0.771^**§**^
*t*_1_	957.5	3518.4	1815.7 (502.0)	1430.8–2157.9	0.000	
FGA						
HGS (kg) *t*_0_	10.7	55.3	28.4 (9.4)	21.7–36.0	0.000	0.000
*t*_1_	10.0	53.7	28.1 (9.3)	21.4–34.5	0.001	
*t*_2_	7.0	48.7	23.8 (9.0)	17.0–30.0	0.000	
TUG (s) *t*_0_	5.0	13.4	8.4 (1.6)	7.3–9.3	0.000	0.000
*t*_1_	5.0	15.2	8.4 (1.6)	7.2–9.0	0.000	
*t*_2_	5.3	15.5	8.9 (1.7)	7.7–9.9	0.000	
SCPT (W) *t*_0_	101.2	456.7	217.2 (49.5)	182.6–243.0	0.000	0.000
*n* = 173						
*t*_1_	102.0	401.8	214.9 (46.5)	183.5–240.9	0.002	
*t*_2_	108.7	368.5	199.8 (45.8)	168.7–224.5	0.000	
4mGS (s) *t*_0_	1.6	4.4	2.8 (0.5)	2.5–3.1	0.000	0.012
*t*_1_	1.7	4.7	2.7 (0.5)	2.4–3.0	0.000	
*t*_2_	1.8	4.5	2.8 (0.5)	2.5–3.1	0.002	
5TCR (s) *t*_0_	6.9	23.1	12.3 (2.9)	10.2–13.5	0.000	0.010
*n* = 172						
*t*_1_	6.3	20.4	11.9 (2.6)	10.0–13.4	0.001	
*t*_2_	6.4	20.2	11.8 (2.6)	10.0–13.3	0.000	
SPPB (pts.) *t*_0_	7	12	11.0 (1.0)	11.0–12.0	0.000	0.176
*t*_1_	8	12	10.9 (1.2)	10.0–12.0	0.000	
*t*_2_	6	12	10.8 (1.3)	10.0–12.0	0.000	
6mWT (m) *t*_0_	250	629	441.2 (67.6)	403.0–479.0	0.146	0.000
*n* = 165						
*t*_1_	250	655	448.3 (67.3)	403.5–485.0	0.101	
*t*_2_	70	610	432.3 (76.9)	395.5–471.5	0.000	

*BMI* body mass index, *FGA* functional geriatric assessment, *HGS* handgrip strength, *TUG* timed up and go test, *SCPT* stair climbing power test, *4mGS* four-meter gait speed, *5TCR* five-times-chair-rise test, *SPPB* short physical performance battery, *6mWT* six-meter walk test

*The Friedman test for *k*-related samples

^$^Shapiro–Wilk test

^**§**^Tests the differences between *t*_0_ and *t*_1_ using the Wilcoxon test

### Sensitivity and specificity

Sensitivity and specificity were calculated as follows: sensitivity = *A*/(*A* + *C*) and specificity = *D*/(*B* + *D*). The development with regard to the delta values of FGA (*t*_0_) to FGA (*t*_2_) was compared using the delta of FGA (*t*_0_) to FGA (*t*_2_) pre-JP (online resource 1).

Outliers were excluded from the analysis. Loss to follow-up occurred and is described in the results.

### Software

Statistical analysis was performed using IBM SPSS Statistics for Windows (IBM Corp. Released 2017, version 26.0, Armonk, NY).

### Reporting

This observational study was reported according to STROBE statement guidelines, provided in the online resource 2 [20].

### Bias

Selection bias may have occurred because cognitively and generally physically fit older adults opted to participate in the study. The study population was identified for a primary prevention study called AEQUIPA (Physical activity and health equity: primary prevention for healthy ageing). In addition, it can be assumed that regular testing of physical functionality motivated the study participants to do additional physical activity to achieve good results.

## Results

A total of 251 participants (mean age 75.4 years) were included in the study; the characteristics of the entire group have been described elsewhere [2]. Four outliers were excluded. Four subjects (1.6%) dropped out of the six-month follow-up, and another 19 (in total *n* = 23 ≙ 9.1%) dropped out of the 24-month follow-up. In eight cases the subject dare to jump actively, due to fear or pain, in two cases the study nurse decided to avoid jumping due to different reasons, e.g. high blood pressure, in 38 cases no CMJ in *t*_0_ or *t*_1_ is present due to different reasons (ground reaction force plate did not work, human error of the study nurse, e.g. did not start system). In total *n* = 75 could not be considered for analysis and *n* = 176 (*n* = 106, 60.2% female, mean age 75.3 (SD 3.7) years) were the maximum for baseline and follow-up data. No differences in characteristics were identified between those individuals who jumped and those who did not jump at all three-time points (see online resource 3).

40.3% (*n* = 71) of subjects increased max JP, 50.6% (*n* = 89) decreased max JP, and 9.1% (*n* = 16) showed changes below the cut-off of 1%, which was defined as data noise and was not considered for further analysis.

The characteristics of the study group including its jump values are presented in Table 2. Except for 6mWT at *t*_0_ and *t*_1_, all variables were not normally distributed (*p* < 0.05). Significant differences according to the Friedman test were identified for the variables of age, HGS, TUG, SCPT, 4mGS, 5TCR, and 6mWT in the follow-up. No changes were found for BMI and SPPB (Table 2).

Table 3 shows Spearman’s correlations between max JP and FGA measurements at *t*_0_ and *t*_1_. The highest correlation for both time points was identified between HGS and max JP, followed by SCPT and max JP (Table 3).

**Table 3:** Correlation coefficient (*r*) of max JP with FGA measurements at baseline (*t*_0_) and at *t*_1_, *n* = 176

	*r* of max JP to—(all *t*_0_)	*r* of max JP to—(all *t*_1_)
HGS	0.736**	0.730**
TUG	−0.095	−0.074
SCPT	0.640**	0.647**
4mGS	0.220**	−0.100
5TCR	0.126	0.135
SPPB	−0.101	−0.087
6MWT	0.275**	0.256**

*Max JP* maximum jumping power, *HGS* handgrip strength, *TUG* timed up and go test, *SCPT* stair climbing power test, *4mGS* four-meter gait speed, *5TCR* five-times chair-rise test, *SPPB* short physical performance battery, *6mWT* six-meter walk test

*< 0.05

**0.01

Table 4 presents predictive data according to the max JP _(0,1)_ model, and shows how these data compare to real measurements (mean %) (Table 4). Here, mean percentage values range from low difference [0.4 (51.3)%] between measured and predicted 4mGS, and increased difference, e.g. between measured and predicted HGS [18.11 (51.9) %], resulting in a difference of 4,65 kg in mean.

**Table 4:** Comparison of FGA measurements at t_2_ to predicted FGA t_2_ according to max JP (*pre-JP*)

*n* = 175	*n*	Min	Max	Mean	SD	Median	1st–3rd quartile	Mean % (SD) of *t*_2_ value
HGS (*t*_2_) pre-JP in kg	176	−7.80	92.12	28.42	16.57	24.50	17.2–39.0	18.11 (51.9)
HGS (*t*_2_) in kg	176	7.00	48.67	23.77	9.04	21.67	17.00–30.00	
TUG (*t*_2_) pre-JP in s	175	−2.5	29.2	8.6	4.7	7.7	5.5–10.6	5.3 (51.6)
TUG (*t*_2_) in s	175	5.3	15.5	8.9	1.7	8.6	7.7–9.9	
SCPT (*t*_2_) pre-JP in W	172	−64.2	731.2	217.6	115.8	199.4	139.3–289.5	8.5 (52.4)
SCPT (*t*_2_) in W	172	108.7	368.5	199.8	45.8	197.9	168.7–224.5	
4mGS (*t*_2_) pre-JP in s	175	−0.8	12.3	2.8	1.6	2.6	2.5–3.1	0.4 (51.3)
4mGS (*t*_2_) in s	175	1.8	4.5	2.8	0.5	2.8	2.0–3.6	
5TCR (*t*_2_) pre-JP in s	172	−4.0	45.8	12.5	7.3	11.1	8.5–15.5	3.7 (52.1)
5TCR (*t*_2_) in s	172	6.4	20.2	11.8	2.6	11.3	10.0–13.3	
SPPB (*t*_2_) pre-JP in pts	175	−2.5	38.0	11.2	5.6	10.7	7.9–14.5	3.1 (51.1)
SPPB (*t*_2_) in pts	175	6.0	12.0	10.8	1.3	11.0	10.0–12.0	
6MWT (*t*_2_) pre-JP in m	164	−97.1	1269.8	451.0	222.9	424.7	308.3–603.7	4.5 (52.6)
6MWT (*t*_2_) in m	164	70.0	610.0	432.5	77.1	435.0	395.3–471.8	

*SD* standard deviation, *FGA* functional geriatric assessment, *HGS* handgrip strength, *TUG* timed up and go test, *SCPT* stair climbing power test, *4mGS* four-meter gait speed, *5TCR* five-times-chair-rise test, *SPPB* short physical performance battery, *6mWT* six-meter walk test

### Clinical relevance

Since mean values are an inadequate way of assessing the health status of subjects in daily clinical routine, sensitivity and specificity of all FGA (*t*_2_) and FGA pre-JP (*t*_2_) are additionally shown in Table 5a–h to give an indication of the clinical impact of the linear prediction of max JP (Table 5). Basis for the calculation of sensitivity and specificity are the CMC or the MDC, which are described in the methods. 5i shows a more general view of sensitivity and specificity. At this point, the tests out of FGA were not compared on test level (e.g. HGS real vs. HGS pre-JP), but more globally as to whether at least one test from the seven of the FGA (1/7) was actually clinically conspicuous with regard to CMC or MDC and at least one of the predicted tests (also 1/7) and the sensitivity and specificity were calculated.

**Table 5:** Cross tables to calculate sensitivity and specificity

a		Groups HGS *t*_2_		Total
		Values < HGS cut-offs	Values > HGS cut-offs	
HGS t_2_ pre-JP	Values < HGS cut-offs	35	15	50
	Values > HGS cut-offs	50	75	125
Total		85	90	175
Sensitivity 41.1%, specificity 83.3%				
b		Groups TUG *t*_2_		Total
		Values > 2.05 s	Values < 2.05 s	
TUG *t*_2_ pre-JP	Values > 2.05 s	3	45	48
	Values < 2.05 s	12	116	128
Total		15	161	176
Sensitivity 20.0%, specificity 72.0%				
d		Groups SCPTP t_2_		Total
		Values < −44 W	Values > −44 W	
SCPTP t_2_ pre-JP	Values < −44 W	3	54	57
	Values > −44 W	2	117	119
Total		5	171	176
Sensitivity 60.0%, specificity 68.4%				
e		Groups 4mGS t_2_		Total
		Values < −0.10 m/s	Values > −0.10 m/s	
4mGS t_2_ pre-JP	Values < –0.10 m/s	7	59	66
	Values > −0.10 m/s	9	101	110
Total		16	160	176
Sensitivity 43.8%, specificity 63.1%				
f		Groups 5TCR *t*_2_		Total
		values < −3.94 s	values > −3.94 s	
5TCR *t*_2_ pre-JP	Values < −3.94 s	3	39	42
	Values > −3.94 s	1	133	134
Total		4	172	176
Sensitivity 75.0%, specificity 77.3%				
g		Groups SPPB t_2_		Total
		Values < 1 point	Values > 1 point	
SPPB *t*_2_ pre-JP	Values < −1 point	27	55	82
	Values > −1 point	29	65	94
Total		56	120	176
Sensitivity 41.5%, specificity 54.1%				
h		Groups 6mWT t_2_		Total
		Values < −50 m	Values > −50 m	
6mWT *t*_2_ pre-JP	Values < −50 m	14	65	74
	Values > −50 m	9	88	102
Total		23	153	176
Sensitivity 60.9%, specificity 57.5%				
i		Overall FGA		Total
		At least 1/7 clinically conspicuous	None	
Overall FGA	At least 1/7 clinically conspicuous	127	43	170
	None	3	3	6
Total		130	46	176
Sensitivity 97.6%, specificity 6.5%				

*FGA* functional geriatric assessment, *JP* jumping power, *HGS* handgrip strength, *TUG* timed up and go test, *SCPT* stair climbing power test, *4mGS* four-meter gait speed, *5TCR* five-time chair rise test, *SPPB* short physical performance battery, *6mWT* six-meter walking test

## Discussion

In the present analysis, we identified the predictive potential of the delta value of two 6-month follow-up measurements of maximum jumping power from the countermovement jump to predict 24-month follow-up FGA measurements of community-dwelling adults above the age of 70 years. The prediction based on the percentage mean value is high, but if sensitivity and specificity are considered to obtain an indication of early primary preventive intervention, maximum jumping power does not seem to be suitable as a predictor in the present cohort.

It has previously been hypothesized that muscle power decreases earlier than strength [11]. As the countermovement jump assesses maximum jumping power, it may be well suited to detect early changes in physical function in adults aged 70 years and older. Maximum jumping power decreased slightly but not significantly in the six-month follow-up period in the present study. In comparison to our study, Singh et al. presented the average value of three jumps and published 945.7 W in men and 700.9 W in women with a mean age of 64.8 for men and 62.5 years for women [17]. In addition to select the mean instead of the maximum value, the study group used another technical device for the measurement, a Tendo power and speed analyzer. In line with the study just mentioned, there is disagreement in the literature as to whether the maximum jump values or average jump values should be used. In addition, various publications calculate jump values related to body weight as well as jump values without any reference. In the present analysis, it was decided to focus on the maximum jumping power of three jumps without body weight adjustment.

Jumping power has already been used to identify the risk of functional deterioration. In Hong et al., the odds of sarcopenia—a common syndrome in older adults—were related cross-sectionally to jumping power; subjects with lower weight-corrected jumping power or those who failed to jump had elevated odds of developing sarcopenia or dysmobility syndrome [21]. In this case, the jumping power seems to be associated with a negative outcome. The observed cohort showed a comparable age (75 years) and was healthy but offers obviously more functional deficits at baseline than our study group, defined by the prevalence of sarcopenia (EWGSOP2) and dysmobility syndrome (consists of, among other, low gait speed, previous falls, low grip strength etc.) of 7.4%, 29.1%.

The InCHIANTI study (*n* = 839, mean age 74.2 years) aimed to identify factors that underlie limitations in mobility among older adults in the community. In particular, it focused on the influences of muscle power and muscle strength on mobility performance (stair climbing, SPPB). Power was not measured by jumping, but via a leg power rig according to Bassey and Short [22]; strength was measured using an isometric dynamometer. The authors concluded that “muscle power identified a more influential proximal determinant of mobility performance than were impairments in strength” [6]. Additionally, in a cohort of 1928 participants from the Toledo Study for Healthy Aging muscle power was assessed by 5-repetition sit-to-stand tests. The group with very low relative power showed higher odds of hospitalization and, as a negative consequence of immobility, all-cause mortality [23]. These studies support using muscle power assessments in older adults such as jumping, which we analyzed.

We provided FGA to measure the physical function of our cohort. We identified a strong correlation between jumping power and HGS, even though HGS represents the strength of the upper extremities. This is in contrast to results from the Osteoporotic Fractures in Men Study (MrOS, *n* = 1242, mean age 84 years), whose subjects with comparable inclusion criteria (≥ 65 years, ability to walk without assistance) showed higher correlations of jumping measures with measures of physical performance (400 m walk time, 6 m gait speed and five times chair rise) than with grip strength. As jumping power had stronger associations than jumping force, which was interpreted as having a stronger relationship between the lower extremities than with the lower to upper extremities [24]. Physical function regarding gait speed and handgrip strength was comparable with the present data (HGS per kg body weight and gait speed of 6 m walking test in m/s, data not shown), but jumping power per kg body weight was higher in the present study (24.5 ± 5.3 vs. 20.8 ± 5.3 in [24]), it may explain the slightly different results between ours and their study.

Our predictive model, which was based on the delta max jumping power values showed a wide range in comparison to the 24-month FGA values (minimal mean % difference was 0.4% (SD 51.3%) in 4mGS and the maximum mean % difference was 18.11% (51.9%) in HGS, Table 4). Solely looking at the mean values may lead to the conclusion that delta jumping power has great potential to predict 24-month follow-up FGA measurements. However, for clinical relevance, we analyzed the sensitivity and specificity of the predicted values which were rather lower than the mean values (sensitivity was low (the maximum value was 75% in 5TCR); specificity was higher [maximum value of 83% (HGS)]. When we did not compare the individual test results with each other, but only looked at whether one of the seven FGA tests showed a clinically conspicuous and also did this with the predicted values, the sensitivity increased as expected. With the help of the jumping power, it seems to be possible with a high sensitivity to predict a clinically relevant change in at least one of the FGA tests and our cohort.

### Strength

The study is a longitudinal cohort study in which the physical functionality of older people aged 70 and over was observed very extensively for an individual period of 2 years. To the best of our knowledge, this is the first analysis to examine the potential of delta maximum jumping power to predict 24-month follow-up FGA measurements.

### Limitations

First, it should be noted that this study is not population based; hence no representation of the general population is given, but rather a selected group of high-performing older adults in the community. To verify that the subjects who jumped do not represent a special group in the overall cohort, the values of those who did not jump were compared to their jumping counterparts with regard to characteristics regarding their age, BMI, and physical function (for baseline, 6-month, and 24-month follow-up); we did not find a clear indication for relevant differences.

As already stated in Diekmann et al. [2], we assume low-level effects due to regular, repetitive geriatric assessments within the study and, in addition, participants may be encouraged to perform well in the study situation. These facts seem to have a positive effect on values within the first 6 months, which may explain why most of the predicted values are overestimated.

We observed some inconsistencies in the three jumps at one measurement time point, outliers were excluded in consequence. In addition, we also measured all jumps using accelerometry. This enabled us to collect data for an evaluation of individual jumps, such as the balance and/or jump phases (drop, jump, landing). Viewed in perspective, sensor-based measurements may offer a better assessment of the risk of falls or loss of mobility. This cohort, which was rather an above-average fit for the age group, showed inconsistencies and a high range of jump values due to individual strategies during the jumping event, such as pulling up the legs in the jumping phase or jumping with the knees straight. In contrast to younger people, older adults rarely jump in their everyday lives. Fear or simply unfamiliar with everyday life may also have affected the target group’s jumping performance.

In addition, only a few subjects fell below the age-associated threshold of FGA (minimal clinically meaningful change or minimal detectable change), which could influence the analysis of sensitivity and specificity of the predicted values. Even if it is an older but fit cohort due to the fact that mainly fit and healthy older individuals tend to participate in research studies, no other, more appropriate tests are available at the moment to identify the risk for functional decline at a very early stage. Therefore we tested the predictive potential of jumping power but as the physician in charge decided against the jump in the last measurement point, only a linear consideration of the two measurement points was possible.

## Conclusion

In the present cohort of high-performing, independent older adults, six-month delta measures of the countermovement jump (maximum jumping power) resulted in a wide range of mean percentage differences (from 0.4% to 18%). With regard to clinical relevance, we focused on sensitivity and specificity. Max JP was not able to predict future functional performance in a 1:1 comparison—in a more globally view considering at least one clinically relevant value out of the seven tests, sensitivity increased significantly.

We assume that also further parameters or combined methods are necessary to evaluate the quality of jumping performance data. In this context, variables such as peak velocity, center of mass, fluctuations, postural sway, and posture control could play a greater role in assessing jumps than power and height alone. A more precise measurement may have the potential to identify vulnerable older adults in a high-performing population. Such identification would enable primary prevention intervention. However, unfortunately the jump may not play a role in the target group, even if the seniors were very fit.

## Supplementary Information

Below is the link to the electronic supplementary material.

Supplementary file1 (DOCX 15 KB)

Supplementary file2 (DOCX 18 KB)

Supplementary file3 (DOCX 16 KB)

## Data Availability

All data generated or analysed during this study are included in this article. Further enquiries can be directed to the corresponding author.
