# Supplementary material for: Are vertical jumps able to predict 24-month follow-up functional geriatric assessment in a healthy community-dwelling older cohort?
Source: Aging Clin Exp Res. 2022 Sep 2;34(11):2769–78. doi: 10.1007/s40520-022-02230-9 (PMC9675680; doi:10.1007/s40520-022-02230-9)
Supplement: Supplementary file 1 — Supplementary file1 (DOCX 15 KB) [file 40520_2022_2230_MOESM1_ESM.docx]

**Supplementary table 1.** Calculation of sensitivity and specificity

|  |  | FGA measurements (t_2_) | |
| --- | --- | --- | --- |
|  |  | **CMC/MDC** | **Normal value** |
| FGA (t_2_) pre-JP | **CMC/MDC**  Positive result | Really positive  A | False positive  B |
|  | **Normal value** Negative result | False negative  C | Really negative  D |
