# Supplementary material for: Are vertical jumps able to predict 24-month follow-up functional geriatric assessment in a healthy community-dwelling older cohort?
Source: Aging Clin Exp Res. 2022 Sep 2;34(11):2769–78. doi: 10.1007/s40520-022-02230-9 (PMC9675680; doi:10.1007/s40520-022-02230-9)
Supplement: Supplementary file 3 — Supplementary file3 (DOCX 16 KB) [file 40520_2022_2230_MOESM3_ESM.docx]

Supplementary Table 3 shows the baseline characteristics and follow-up characteristics of subjects who did not perform the CMJ at baseline and at t_1_.

**Supplementary Table 3.** Baseline (t_0_) and follow-up (t_1_, t_2_) characteristics of non-jumpers

|  | **n (%)** | **min** | **max** | **mean (SD)** | **1st to 3rd quartile** | **p-value*** |
| --- | --- | --- | --- | --- | --- | --- |
| Age [years] *t_0_* | 51 | 70 | 86 | 75.2 (4.1) | 72.0-77.3 | 0.469 |
| *t_1_* | 47 | 70 | 86 | 75.8 (4.1) | 73.0-77.3 | 0.454 |
| *t_2_* | 38 | 71 | 88 | 77.1 (4.1) | 74.0-79.3 | 0.392 |
| Female *t_0_* | 36 (56.3) |  |  |  |  |  |
| BMI [kg/m^2^] *t_0_* | 51 | 20.4 | 38.8 | 28.4 (4.2) | 25.9-30.5 | 0.071 |
| *t_1_* | 47 | 20.2 | 39.5 | 28.2 (4.1) | 27.3-30.1 | 0.071 |
| *t_2_* | 38 | 19.5 | 38.7 | 28.3 (4.1) | 25.7-29.9 | 0.091 |
| ***Physical function*** |  |  |  |  |  |  |
| HGS [kg] *t_0_* | 51 | 12.7 | 53.7 | 30.6 (10.8) | 21.8-40.4 | 0.720 |
| *t_1_* | 47 | 12.0 | 50.0 | 30.2 (10.6) | 21.8-38.3 | 0.448 |
| *t_2_* | 38 | 8.7 | 47.7 | 25.9 (11.0) | 16.8-36.2 | 0.249 |
| SCPT [W] *t_0_* | 51 | 136.7 | 339.3 | 230.9 (49.8) | 195.6-270.2 | 0.326 |
| *t_1_* | 47 | 139.6 | 347.2 | 233.5 (49.9) | 192.4-273.9 | 0.109 |
| *t_2_* | 38 | 78.7 | 320.20 | 213.5 (53.3) | 177.3-247.8 | 0.083 |
| TUG [s] *t_0_* | 51 | 6.0 | 14.1 | 8.5 (1.7) | 7.4-9.2 | 0.958 |
| *t_1_* | 47 | 5.5 | 15.1 | 8.5 (1.7) | 7.2-9.4 | 0.694 |
| *t_2_* | 38 | 6.3 | 16.6 | 9.1 (2.3) | 7.3-10.1 | 0.610 |
| 4mGS [s] *t_0_* | 51 | 1.8 | 4.3 | 2.7 (0.5) | 2.4-3.0 | 0.529 |
| *t_1_* | 47 | 1.8 | 4.5 | 2.7 (0.5) | 2.4-3.0 | 0.780 |
| *t_2_* | 38 | 1.9 | 5.9 | 2.8 (0.7) | 2.4-3.1 | 0.477 |
| 5TCR [s] *t_0_* | 51 | 6.7 | 21.0 | 12.2 (2.8) | 10.7-13.8 | 0.870 |
| *t_1_* | 47 | 7.4 | 21.4 | 11.7 (3.3) | 9.3-14.1 | 0.197 |
| *t_2_* | 38 | 7.5 | 20.3 | 11.2 (2.7) | 9.4-12.8 | 0.259 |
| SPPB [pts.] *t_0_* | 51 | 8.0 | 12.0 | 11.0 (1.1) | 10.0-12.0 | 0.788 |
| *t_1_* | 47 | 9.0 | 12.0 | 11.0 (1.1) | 10.0-12.0 | 0.807 |
| *t_2_* | 38 | 9 | 12 | 10.9 (1.6) | 9.0-12.0 | 0.315 |
| 6mWT [m] *t_0_* | 51 | 198.0 | 589.0 | 435.8 (82.4) | 377.5-500.0 | 0.800 |
| *t_1_* | 47 | 245.0 | 611.0 | 442.6 (85.7) | 393.8-496.3 | 0.773 |
| *t_2_* | 38 | 109 | 562 | 420.5 (88.2) | 366.5-465.8 | 0.402 |

* The Mann–Whitney U tests jumpers vs. non-jumpers, n=50 vs. 176 at t_0_, t_1_, and t_2_.
